# The Impact of Endometriosis across the Lifespan of Women: Foreseeable Research and Therapeutic Prospects

**DOI:** 10.1155/2015/158490

**Published:** 2015-05-06

**Authors:** C. L. Hughes, W. G. Foster, S. K. Agarwal

**Affiliations:** ^1^Cardiovascular & Metabolic Diseases, Medical Strategy & Science, Quintiles, Inc., 5927 S. Miami Boulevard, Morrisville, NC 27560, USA; ^2^Department of Obstetrics and Gynecology, Duke University Medical Center, Durham, NC 27710, USA; ^3^Department of Obstetrics and Gynecology, McMaster University, Hamilton, ON, Canada L8S 4L8; ^4^Department of Reproductive Medicine, University of California-San Diego, San Diego, CA 92093-0633, USA

## Abstract

In addition to estrogen dependence, endometriosis is characterized by chronic pelvic inflammation. The impact of the chronic pelvic inflammatory state on other organ systems and women's health is unclear. Endometriosis associated chronic inflammation and potential adverse health effects across the lifespan render it imperative for renewed research vigor into the identification of novel biomarkers of disease and therapeutic options. Herein we propose a number of opportunities for research and development of new therapeutics to address the unmet needs in the treatment of endometriosis *per se* and its ancillary risks for other diseases in women across the lifespan.

## 1. Introduction

Although a number of causal or exacerbatory pathological mechanisms seem to underlie initiation and persistence or progression of endometriosis [1–12] there is no debate that this disease features a chronic inflammatory state within the peritoneal cavity [9, 10]. The extent to which this disease process impacts the health of women surely relates to inflammation within this anatomical/physiological compartment. As a corollary, it is plausible that systemic chronic inflammatory effects also result and endometriosis could induce adverse effects on other tissues or body systems. Given the broad and widely accepted notion that chronic inflammation is a risk factor for various degenerative or “Western Diseases” such as atherosclerosis, multiple cancers, dementias, degenerative joint diseases, inflammatory bowel diseases, and others, endometriosis must be considered as a potential risk factor for a spectrum of other diseases that may adversely impact the health of women across the lifespan. If one or more such associations are established and one or more causal relationships can be demonstrated, then medical care of these young adult patients will be driven by additional imperatives that will extend far beyond those extremely important health impacts that are currently recognized related to loss of fertility, debilitating pain, and destruction of urogenital and gastrointestinal tissues and organs. Our endometriosis patients deserve insightful and expert care for all of the potential types of harm that this disease can incur. From this perspective, we will summarize or propose a number of opportunities for research and development of new therapeutics to address the unmet needs in the treatment of endometriosis* per se* and its ancillary risks for other diseases in women across the lifespan.

## 2. Causality in the Association of Endometriosis and Intraperitoneal Inflammation

The association of endometriosis with intraperitoneal inflammation is thoroughly acknowledged by physicians and investigators in this field. The causal relationship regarding which comes first may not be absolutely established [3] but research with nonhuman primate models strongly supports the contention that the initiation of endometriosis implants triggers the inflammatory effects rather than the other way around [13]. It is quite possible that endometriosis is both the result of and the cause of further inflammation.

## 3. Key Issues in Understanding and Managing the Effects of Endometriosis across the Lifespan

No matter whether the disease etiology depends primarily upon the biomechanics of retrograde menstruation, sex hormone modulation of endometrial tissue proliferation, environmental exposures, genetics, oxidative stress, or inflammatory cell populations, we must address three key themes:

1. we must develop novel biomarkers of endometriosis for diagnosis, response to treatment, and disease progression;
2. we must ascertain whether the chronic inflammatory process in the peritoneal compartment incurs a significant risk for other systemic (remote) diseases [14–18];
3. we must strive to identify novel preventative, modulatory, or therapeutic interventions that can take advantage of cellular and molecular mechanisms to mitigate both the primary disease process (intraperitoneal endometriosis) and the consequent systemic inflammatory effects.

## 4. Innovations in the Search for a Biomarker of Endometriosis

A plethora of biochemical differences in the peripheral circulation, peritoneal fluid, and endometrial tissues of women with endometriosis versus healthy controls has been demonstrated [40–42] many of which are related to a chronic inflammatory reaction [43–50]. Other biomarkers that have been examined include vascular endothelial growth factor (VEGF) [51–53], glycodelin [54–56], different biomarkers in the apoptosis pathway including the annexin family [57–59], and soluble intracellular adhesion molecule-1 [60–63]. Of the vast number of factors that have received attention as potential diagnostic biomarkers of endometriosis, cancer antigen 125 (CA125) is potentially the most widely studied [46, 64–66]. However, use of CA125 as a single diagnostic biomarker of endometriosis is unacceptable owing to low sensitivity [67]. While the search for clinically useful markers of endometriosis continues, there is growing evidence that a compact panel of molecular markers may show the performance characteristics needed to serve as a practical screening or diagnostic test, especially if used as part of a multiparameter mathematical model [50, 68–70].

Emerging areas of interest include nerve fiber density, microRNA (miRNA), and neurotrophins. Recent studies report the fact that nerve fiber density in the functional layer of the eutopic endometrium is greater in women with endometriosis compared to controls [71, 72]. Although this conclusion was recently challenged [73], the measurement of nerve fiber density has been put forward as a diagnostic tool for mild to minimal endometriosis [74]. Unfortunately, measurement of nerve fiber density requires an endometrial biopsy and thus is more technically demanding, painful, time consuming, and resource intensive than a simple blood test and is therefore potentially less appealing to women and their health care providers. In contrast, mean plasma concentrations of the neurotrophin, brain derived neurotrophic factor (BDNF), were greater than 2 times higher in women with endometriosis versus healthy asymptomatic controls whilst no differences were found at three months after surgical removal of lesions [75]. Moreover, increased expression of BDNF and neurotrophin 4/5 has been demonstrated in the endometrium of women with endometriosis versus healthy controls [76]. Taken together these studies suggest that the neurotrophins could hold promise as biomarkers of endometriosis.

Several recent studies have documented aberrant expression of several different miRNAs in women with endometriosis [77–82]. miRNAs are short noncoding RNAs that negatively regulate mRNA translation by repressing the protein translational machinery or degrading their target transcripts. Greater than 2000 mature human miRNA sequences have been identified and are thought to regulate approximately 50% of all protein coding genes. Although widely studied in cancer, the role of miRNAs in regulation of proteins important in the pathophysiology of endometriosis is relatively unexplored. Unfortunately, we could find no agreement amongst the miRNA changes documented in the studies conducted to date [77–82] which we suggest arises from differences in tissues sampled (deep infiltrating, peritoneal, or endometrioma), burden of disease, type of lesion (red, blue-black versus white), and failure to adequately control for confounding factors [83].

Prospects for development of improved diagnostic and disease-monitoring strategies for endometriosis have recently been advanced by the efforts of a major international research consortium, the international “WERF EPHect” initiative [84–88]. In this important research program, major strides have been made regarding the bases for harmonizing the way data, surgical phenotype, tissues, and fluid samples are collected and should serve as a critical basis for more coherent future research in this field.

## 5. Evidence Regarding Systemic Inflammation and Disease Associations in Women with Endometriosis

Contemporary biomarker techniques [17] are beginning to demonstrate the range of molecules that can be detected systemically in women with endometriosis that are plausibly related to the range of autoimmune and other inflammatory disorders that have been associated by survey research [89]. A modest number of studies have been published suggesting that risk factors for and an increased occurrence of a number of chronic diseases occur in women with endometriosis [90–94]. The range of associated diseases is relatively wide but largely hinges on the concept that chronic inflammation and oxidative stress are systemically increased in the women with endometriosis and these pathways are a plausible basis for such associations and perhaps in due course evidence of causation. Endometriosis is not the only reproductive tract related inflammatory process that has been implicated in the risks associated with systemic inflammation since endothelial dysfunction has also been demonstrated in men with chronic prostatitis and chronic pelvic pain syndrome [95]. The impact of benign but chronic reproductive tract diseases in both sexes merit committed research and development of safe and effective therapeutics.

## 6. Unique Considerations for the Therapeutic Challenge When Considering the Impact of Endometriosis across the Lifespan

Current therapeutic drugs are primarily directed at suppressing the intraperitoneal activity of the underlying disease. As is the case for other chronic diseases, future novel therapeutics for endometriosis will need to be developed to treat the primary intraperitoneal process [20], the potential associated intraperitoneal malignant risk(s) [96, 97], and the potentially distant disease risk factors [98]. New lifespan therapies that treat either the primary intraperitoneal process or both endometriosis* per se* and systemic adverse effects with durability will mean either long-term continuing usage or repetitive episodic usage with convincing evidence of patient safety and tolerability. Accordingly investigators in this field need to consider the prospects for patient safety with chronic usage of any candidate agents along with evidence of efficacy and reduction in risks of other diseases associated with chronic inflammation [99]. Whether diet is considered broadly [100] or in terms of specific food groups [101] aimed at reduction of risks of multiple chronic diseases, humans in general do not consume the quantities of vegetables and fruits that would afford beneficial effects as seen with higher levels of healthful protective dietary phytochemicals. For more targeted management of endometriosis, we should study dietary (nutritional and functional) interventions, generally recognized as safe (GRAS) compounds, and repurposing of established pharmaceuticals as well as novel compounds for efficacy but all must also be assessed with this long-term safety imperative in mind.

Beyond the current set of hormonal and anti-inflammatory drugs used to treat endometriosis, what classes of compounds might be safe and effective in the sustained management of this chronic inflammatory process? A modest number of ideas have been suggested by various investigators and we shall consider them plus one new idea that we propose from some recent research in continuous ambulatory peritoneal dialysis (CAPD).

Several classes of compounds (Table 1) have been or could be directed toward development as therapeutics for the intraperitoneal compartment aspects of endometriosis [1, 19–30]. Several of these would modify inflammation or oxidative stress by one mode of action or another while others could be developed to affect implantation and survival of endometriosis implants or to mitigate some of the actions of cellular mediators of endometriosis associated inflammation.

The second group of compounds that merit research and development for potential application in endometriosis may potentially mitigate both the intraperitoneal and the systemic inflammatory consequences of endometriosis [20, 34–39]. On one hand, the recent evidence that omega-3 fatty acids may have anti-inflammatory effects in monocytes by acting on the GPR120 receptor [38] raises the attractive prospect that a dietary or nutritional supplement could be shown to benefit women with endometriosis. The opportunity to repurpose or extend the indications for well-known pharmaceuticals [20, 34, 35] to use in the long-term care of women with endometriosis could also offer options for subpopulations of patients who have concomitant disorders such as dyslipidemia or glucose intolerance/type II diabetes mellitus whether we are or are not able to attribute their metabolic disorders to the presumed risk of antecedent or ongoing endometriosis. Another drug repurposing prospect is the gout therapy, colchicine. This well-known anti-inflammatory drug has been used for decades and has a well-established safety record when used chronically to suppress recurrent flares of gout [37]. Additionally, a recent study in an animal model of endometriosis showed colchicine to be highly effective in suppressing intraperitoneal implant volumes and concentrations of TNF-*α* in peritoneal fluid [38]. Finally, based on a new study in an animal model of continuous ambulatory peritoneal dialysis (CAPD) in which oral administration of spironolactone was remarkably effective at decreasing intraperitoneal fibrosis and inflammation [36] and since reproductive endocrinology and infertility subspecialists have substantial experience with the safe chronic use of spironolactone in women with androgen excess disorders, we urge investigators to consider research to assess the therapeutic potential of spironolactone or other aldosterone receptor antagonists in endometriosis.

## 7. Summary and Prospects for Future Therapeutic Developments

It is our view that there are ample opportunities to pursue research regarding the impact of endometriosis across the lifespan and to pursue development of a number of therapeutics that could plausibly be expected to be both safe and beneficial for women suffering with this disease. This is not an argument for taking our eye off the prize meaning endometriosis as the primary disease; rather we owe it to each of our patients to consider all of the impacts at all times in life that this disease may incur. We urge deeper and broader understanding of such risks and pursuit of safe and effective interventions for the care of our patients.

## Figures and Tables

**Table 1:** Possible future therapeutics for endometriosis and endometriosis-related systemic inflammation.

Representative compound	Class/category	Mode(s) of action	Representative references

Intraperitoneal compartment: disease process and inflammation			
Pycnogenol	Diet/supplement	Nuclear factor-kappa B inhibitor	[19]
Novel compounds	Pharmaceuticals	Angiogenesis inhibition	[20, 21]
Resveratrol, other novel compounds	Diet/supplement or pharmaceuticals	Sirtuin 1 pathway/anti-inflammatory	[22]
Melatonin	Supplement	Antioxidant	[23–25]
Novel compounds, vitamin D/analogues	Pharmaceuticals or vitamin D plus progesterone	Semaphorins and plexins/cell attachment	[26–29]
Novel biopharmaceutical compounds	siRNA	Inflammatory monocytes	[30]
AH6809, AH23848	Prostaglandin E2 receptor (PTGER2 or PTGER4) antagonists	Prostaglandin E2 antagonism	[31–33]
Novel compounds	Histone deacetylase inhibitors	Epigenetic modification	[1]

Intraperitoneal and systemic inflammation: interface between compartments			
Statins	Well-known pharmaceuticals	Inhibition of HMG-CoA reductase and anti-inflammatory	[20, 34]
Metformin	Well-known pharmaceutical	Anti-inflammatory	[35]
Omega-3-fatty acid, novel compounds	Diet/supplement or pharmaceuticals	Anti-inflammatory and inflammatory monocytes via GPR120	[36]
Spironolactone (recent study in CAPD)	Well-known pharmaceutical	Intraperitoneal and systemic inflammatory via aldosterone receptor antagonism	[37]
Colchicine	Well-known pharmaceutical	Intraperitoneal and systemic inflammatory via multiple modes of action	[38, 39]
